# Spontaneous adverse drug reaction reporting: potential facilitators perceived by community pharmacists in Egypt – a cross-sectional study

**DOI:** 10.1007/s44446-025-00025-3

**Published:** 2025-09-22

**Authors:** Mohamed Bahlol, Mary Bushell, Hani M. J. Khojah, Rebecca Susan Dewey

**Affiliations:** 1 Specialty of Pharmaceutical Management and Economics, Department of Pharmacy Practice and Clinical Pharmacy, Faculty of Pharmacy, Egyptian Russian University, Badr City, Cairo Governorate, Egypt; 2 Discipline of Pharmacy, Faculty of Health, University of Canberra, Bruce, Canberra, Australia; 3 Department of Clinical and Hospital Pharmacy, College of Pharmacy, Taibah University, Madinah, Kingdom of Saudi Arabia; 4 Sir Peter Mansfield Imaging Centre, School of Physics and Astronomy, University of Nottingham, Nottingham, UK

**Keywords:** Adverse drug reactions, ADR reporting, Community pharmacists, Pharmacovigilance, Medication safety, Egypt

## Abstract

Pharmacists are recognized as specialists in medications and are responsible for maintaining drug safety. A recent study showed that Egyptian community pharmacists face several barriers to the spontaneous reporting of adverse drug reactions (ADRs). This study aimed to identify the potential facilitators perceived by community pharmacists in Egypt that could enhance ADR reporting and contribute to the development of national ADR data. A cross-sectional survey was conducted using a self-administered questionnaire distributed to 1,316 community pharmacists in Egypt. Of the 905 respondents (68.7% response rate), only 125 (13.8%) revealed they had reported an ADR, with 30 (24.4%) being unable to correctly identify ADR types and 34 (27.2%) reporting they lacked the training required to do so. Key facilitators identified be respondents included ensuring that ADR-related training is available from universities (95.7%), the Egyptian Pharmacists Syndicate (91.9%), and peer-reviewed journal articles (90.6%). Participants advocated for simplifying the reporting process (92.5%), providing clear instructions (92.8%), having access to a smartphone application (80.0%), receiving regular reminders (92.4%) having their role promoted in the media (95.6%). Community pharmacists are crucial in ADR reporting, especially in low-to-middle-income countries e.g. Egypt. The study identified several facilitators to improve reporting practices, including educational interventions, process enhancements, and motivational strategies. Implementing these facilitators could address underreporting and data inaccuracies.

**Supplementary information:**

The online version contains supplementary material available at 10.1007/s44446-025-00025-3.

## Introduction

Medicinal products play a crucial role in preventing and treating diseases, improving health and wellbeing, and saving lives. However, they are not entirely without risk (Insani et al. 2021). According to the World Health Organization (WHO), an adverse drug reaction (ADR) is defined as *“a response to a drug that is noxious and unintended and occurs at doses normally used in man for the prophylaxis, diagnosis or therapy of disease, or for modification of physiological function” (*World Health Organization 1972*).* ADR reporting can predict potential hazards from future administrations, necessitating prevention, dosage adjustments, or the withdrawal of the product (Aronson 2023). ADRs can range from mild to life-threatening and are a significant cause of hospitalization and mortality in both developed and developing countries (Patel and Patel 2018).

Despite the rigorous clinical trial process for novel medicines, not all ADRs are identified before a drug is approved and released to the market (Berlin et al. 2008). Therefore, a drug's safety continues to be monitored after it becomes widely available to the public. This post-marketing surveillance is the cornerstone of pharmacovigilance, helping to identify ADRs in real-world settings and more diverse populations, as well as establishing safety data for long-term use.

Pharmacovigilance primarily relies on spontaneous reporting of ADRs to local authorities (Alomar et al. 2020). Most high-income developed countries and an increasing number of low-to-middle-income countries have pharmacovigilance systems managed by the respective medicines regulatory body or government agency, enabling the identification and evaluation of previously unreported and unknown ADRs (Alshammari et al. 2019).

In Egypt, the Ministry of Health and Population (MOHP) and the Egyptian Drug Authority (EDA) established the Egyptian Pharmaceutical Vigilance Center (EPVC) in 2009 (Bahlol et al. 2022). The EPVC collects, collates, and analyzes all ADR reports in the national database. When necessary, it alerts health professionals, manufacturers, and the public to new ADR risks. As a member of the WHO Programme for International Drug Monitoring (PIDM), the EPVC also submits reports to the WHO global database (World Health Organization 2023).

ADR reports can be submitted to the EPVC either in writing or electronically, directly by patients (or their caregivers) or indirectly by healthcare providers such as nurses, pharmacists, and physicians (Egyptian Pharmaceutical Vigilance Center 2023). Despite the EPVC’s 15-year establishment and encouragement of relevant professionals to report suspected ADRs, underreporting remains a challenge, as it is in other countries (Elsayed and Al-Worafi 2020; Aziz et al. 2022).

Pharmacists, being experts in medicinal products and custodians of their safety, have regular contact with patients and are ideally positioned to detect and report ADRs (International Pharmaceutical Federation 2021) (Cheema et al. 2017). This is especially pertinent in low-to-middle-income countries where community pharmacies are widely distributed. A recent systematic review highlighted that ADRs are a significant problem in the primary care setting (Insani et al. 2021). Therefore, community pharmacists are key stakeholders in increasing the detection and reporting of ADRs. Given Egypt’s high number of pharmacies per capita (6.5 per 10,000 people), pharmacists are expected to understand and report ADRs to contribute to the safe and effective use of medicines. However, a cross‐sectional survey in Egypt identified numerous barriers to community pharmacist-led reporting (Bahlol et al. 2022). Nevertheless, those barriers contribute to the problem of underreporting; facilitators have not yet been reported within the setting of Egyptian community pharmacies. Thus, this study aimed to quantitatively address the following question: *What are the potential facilitators perceived by community pharmacists that can enhance ADR reporting and the development of national ADR data?*

## Methods

### Study design

**Fig. 1:**
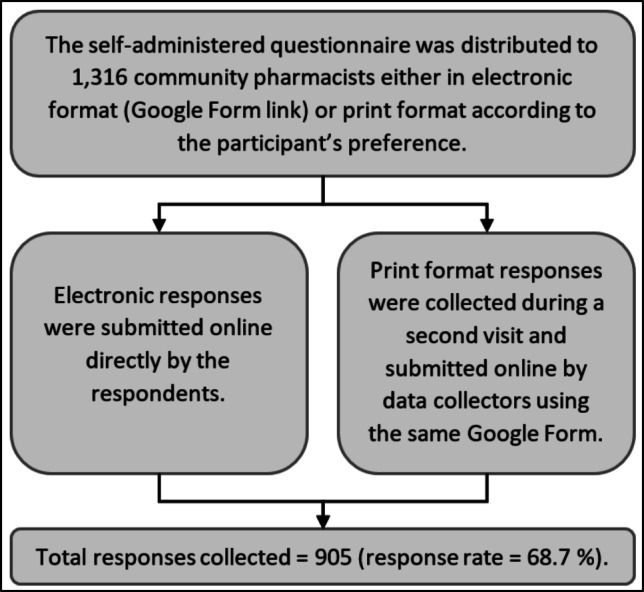
Data collection flowchart

A cross‐sectional survey was conducted in April 2024 using a self-administered questionnaire to capture the views and experiences of community pharmacists (Fig. 1). Data collectors were recruited to visit selected pharmacies and distribute questionnaires either as printed forms or online links to electronic forms, depending on the pharmacist’s preference. This drop-and-collect technique usually yields a higher response rate and reduces selection bias (Walker 1976; Brown 1987). One pharmacist from each visited pharmacy was asked to respond.

### Ethical considerations

The study adhered to the guidelines of the Declaration of Helsinki (World Medical Association 2013) and was approved by the Institutional Review Board of the Faculty of Pharmacy, Egyptian Russian University (approval no. ERUFP-PP-17–001). A confidentiality statement was included in the survey form, and all participants provided informed consent.

### Data collectors

A total of 200 trained pharmacy students were recruited to distribute and collect the forms from community pharmacists within their geographical regions. Participation of trained undergraduate students in field studies is a component of the pharmacy curriculum in Egypt, especially in courses on pharmaceutical management and economics. Consistent with the National Authority for Quality Assurance and Accreditation of Education and the Egyptian Supreme Council of Universities, primary educational objectives include professional growth and connecting students to the real-world environment of the labor market (Egyptian Ministry of Higher Education and Scientific Research. Higher Education Strategy 2020; Egyptian National Authority for Quality Assurance and Accreditation of Education 2020). The students were instructed to adhere to preventive measures such as wearing face masks to prevent the spread of infectious respiratory diseases, including COVID-19, during pharmacy visits.

### Questionnaire development

To ensure a broad and comprehensive context for the domain under investigation and to minimize potential bias, the questionnaire was developed based on an extensive review of relevant global studies (Oвчинникoвa E. A. [Ovchinnikova E. A.] 2003, Alraie et al. 2016, Ampadu et al. 2016, Li et al. 2018, Vuković Rodríguez and Juričić 2018, Bahlol and Dewey 2021). Additionally, the opinions of twelve highly experienced local pharmacists were sought to ensure face and content validity of the questionnaire before the pilot test period.

### Pilot test

A pilot test was conducted on a random sample of 36 pharmacies. During this phase, feedback was requested from the pharmacists regarding the structure and content of the forms, which led to several amendments, including modifications to questions and sentence formatting. All versions of the questionnaire were produced in the local Arabic language.

### Questionnaire content

The questionnaire consisted of two sections with closed-ended questions. The first section collected sociodemographic information, while the second section focused on the domain under investigation, namely the “potential facilitators” (subdomains: educational intervention, motivating factors, and the reporting process itself) for ADR reporting. The internal consistency of the questionnaire was measured using Cronbach's alpha (α = 0.78). The English translation of the questionnaire is provided in the Supplementary material.

### Pharmacy selection

There are approximately 70,000 registered community pharmacies in Egypt (Bahlol and Dewey 2021). The sample size was calculated using the formula X = Z_α/2_^2^ *p*(1-p)/MOE^2^, where Z = 2.576, confidence level = 99%, margin of error = 5%, and sample proportion = 50% (Daniel and Cross 2018). This yielded a minimum sample size of 658, with 90% power (Friedman 1982; Ozdimer et al. 2006). Pharmacies were selected using stratified random sampling at a regional level, covering the seven regional units across Egypt: Center (Greater Cairo), North (Delta and Alexandria), South (North, Assiut/Center, and South of Upper Egypt), and East (Suez Canal) (General Organization for Physical Planning 2020).

### Data analysis

All completed response forms were coded before analysis to keep the data analyzer blinded to respondent identity. Analyses were conducted using SPSS version 20. Descriptive statistics and comparative analyses between survey items were performed. To display the average trends of the group data, means (± SD) were calculated. To assess the relationships between specified variables and demographic characteristics, Pearson’s chi-square tests were employed, with p ≤ 0.05 considered significant. For transparency, and due to the presence of a small number of missing values in the data, the tabulated results show both absolute numbers and the percentage of valid responses.

## Results

### Demographics

A total of 1,316 pharmacists were contacted (more than double the minimum required sample size of 658), and of these, 905 participated, making a response rate of 68.7%. Questionnaires with incomplete responses (i.e., missing data) were included in the statistical analysis, with each variable of interest lacking between 1 and 6 responses (mode = 2; mean = 1.72) of missing data (Table 1).

**Table 1:** Respondents’ demography (*N* = 905)

Characteristics	*n*	Valid %
**Region**		
* South*	59	6.5
* East*	66	7.3
* Centre*	156	17.3
* North*	622	68.9
* Missing*	2	-
**Position**		
* Junior*	301	33.4
* Senior*	200	22.1
* Registered manager*	402	44.5
* Missing*	2	-
**Graduation year**		
* Mean* + *SD* = *2006* ± *10.35*		
* Median (range)* = *2008 (1972–2022)*		
* Missing*	4	-
**Experience in community setting (years)**		
* Mean* + *SD* = *13* ± *9.84*		
* Median (range)* = *10 (1–50)*		
* Missing*	5	-
**Sex**		
* Male*	661	73.2
* Female*	242	26.8
* Missing*	2	
**Age (years)**		
* Mean* + *SD* = *36* ± *10.57*		
* Median (range)* = *34 (23–75)*		
* Missing*	4	-
**Type of university studied at**		
* Governmental*	751	83.2
* Private*	152	16.8
* Missing*	2	-
**Correctly identified ADR types**		
* No*	318	35.3
* Yes*	582	64.7
* Missing*	5	-
**Had received training on ADR reporting**		
* No*	543	60.1
* Yes*	360	39.9
* Missing*	2	-
**Preferred paper-based reporting (Yellow Card)**		
* No*	731	80.9
* Yes*	173	19.1
* Missing*	1	-
**Preferred electronic reporting (online)**		
* No*	625	69.3
* Yes*	277	30.7
* Missing*	3	-
**Involved in ADR reporting**		
* No*	780	86.2
* Yes*	125	13.8
* Missing*	0	-

*ADR* adverse drug reaction, *SD* standard deviation

The experience of pharmacists in the sample ranged from 1 to 50 years in community settings, covering all positions; juniors (301, 33.4%), seniors (200, 22.1%), and registered managers (402, 44.5%) across all regions of the country; South (59, 6.5%), East (66, 7.3%), Central (156, 17.3%), and North (622, 68.9%).

### Reporting practices

Only 125 surveyed pharmacists stated that they had reported ADRs. However, 30 of these reporters (24.4%) could not correctly identify ADR types such as non-dose-related (bizarre), dose-related (augmented), time-related (delayed), dose-related and time-related (chronic), failure of therapy (failure), and withdrawal (end of use) (Edwards and Aronson 2000). Moreover, 34 pharmacists (27.2%) reported they had not received training on ADR reporting (Table 2). The information provided to pharmacists by customers and the demography of the reporting pharmacists are given in Table 3 and Table 4, respectively.

**Table 2:** Pharmacists’ reporting practices (*N* = 125)

Reporting practice	*n*	Valid %
Correctly identified ADR types^*^		
* No*	30	24.4
* Yes*	93	75.6
* Missing*	2	-
**Preferred paper reporting (Yellow Card)**		
* No*	69	55.2
* Yes* * Missing*	56 0	44.8 -
**Preferred electronic reporting (online)**		
* No*	51	40.8
* Yes* * Missing*	74 0	59.2 -
**Had received training on ADR reporting**		
* No*	34	27.2
* Yes* * Missing*	91 0	72.8 -

*ADR* adverse drug reaction

**Table 3:** Adverse drug reaction information presented by customers (*N* = 125)

Information	*n*	Valid %
**Severity of symptoms**		
* Mild*	21	16.8
* Moderate*	40	32.0
* Severe*	5	4.0
* Mild and moderate*	21	16.8
* Mild and severe*	3	2.4
* Moderate and severe*	1	0.8
* Mild, moderate, and severe* * Missing*	34 0	27.2 -
**Types of drugs**		
* Prescription*	22	17.6
* Over the counter*	5	4.0
* Both* * Missing*	98 0	78.4 -
**Sources of drugs**		
* Synthetic*	41	32.8
* Natural*	4	3.2
* Both* * Missing*	80 0	64.0 -
**Patients’ ages**		
* Adults*	16	12.8
* Children*	3	2.4
* Both* * Missing*	106 0	84.8 -

**Table 4:** Demography of the reporting pharmacists (*N* = 125)

Information	*n*	Valid %
**Region**		
* South*	5	4.0
* East*	12	9.6
* Center*	28	22.4
* North* * Missing*	80 2	64.0
**Position**		
* Junior*	43	35.0
* Senior*	35	28.5
* Registered manager*	45	36.5
* Missing*	2	-
**Graduation year**		
* Mean* + *SD* = *2005.29* ± *9.75*		
* Median (range)* = *2007 (1980–2019)*		
* Missing* = *4*		
**Experience in community setting (years)**		
* Mean* + *SD* = *13.61* ± *8.91*		
* Median (range)* = *11 (1–40)*		
* Missing* = *5*		
**Sex**		
* Male*	85	68.0
* Female* * Missing*	40 2	32.0
**Age (years)**		
* Mean* + *SD* = *37.19* ± *9.75*		
* Median (range)* = *35 (23–62)*		
* Missing* = *4*		
**Type of university studied at**		
* Governmental*	108	86.4
* Private*	17	13.6
* Missing*	10	-

*SD* standard deviation

### Facilitators for reporting

#### Educational intervention

Respondents emphasized the need for enhancing knowledge and skills through university teaching about the reporting process (866, 95.7%) and ADRs (976, 97.0%) (Tables 5A and 6A). They also highlighted the importance of continuing professional development (CPD) provided by the Egyptian Pharmacists Syndicate (EPS) (832, 91.9%) and online access to trusted, peer-reviewed scientific journal articles (820, 90.6%).

**Table 5:** Potential facilitators to adverse drug reaction reporting (N = 905)

Facilitators	n	(%)^a^	Region (%)^b^					Position in pharmacy (%)^b^				University of graduation (%)^b^			Graduation year	Sex (%)^b^		
			South	East	Center	North	*P*^*c*^	Junior	Senior	Manager	*P*^*c*^	Government	Private	*P*^*c*^	*P*^*c*^	Male	Female	*P*^*c*^
**A. Educational intervention**																		
University teaching about the reporting process	866	95.7	93.2	97.0	96.2	95.7	NS	97.0	95.0	95.0	NS	95.5	96.7	NS	NS	95.6	95.9	NS
University teaching about ADRs	876	97.0	93.2	98.5	96.1	97.4	NS	96.0	97.5	97.5	NS	97.1	96.7	NS	NS	96.7	97.9	NS
CPD delivered by the EPS	832	91.9	91.5	90.9	87.2	93.2	NS	89.4	91.5	94.0	NS	91.6	93.4	NS	NS	92.1	91.3	NS
CPD delivered by the health directorate	766	84.7	87.9	81.8	81.4	85.5	NS	77.7	87.0	88.8	< 0.001	83.7	89.5	NS	NS	84.4	85.5	NS
CPD delivered by a university	752	83.2	87.9	87.9	75.6	84.1	0.034	80.3	85.5	84.1	NS	83.2	82.9	NS	0.034	83.8	81.4	NS
CPD delivered by pharmaceutical companies	788	87.1	91.5	89.4	87.8	86.2	NS	86.7	87.0	87.3	NS	86.4	90.1	NS	NS	86.8	87.6	NS
Peer-reviewed online journal articles	820	90.6	89.8	93.9	92.3	89.9	NS	89.7	92.0	90.5	NS	90.8	90.1	NS	0.005	90.5	90.9	NS
Mean ± SD: *n* = *814.3* ± *47.8* *%* = *90.0* ± *5.3*																		
**B. The reporting process**																		
A smartphone application for reporting	724	80.0	72.9	81.8	83.3	79.6	NS	81.4	84.5	76.6	NS	79.2	84.2	NS	0.004	79.0	83.1	NS
Easily accessible Yellow Cards	590	65.3	62.7	69.7	69.0	64.3	NS	66.4	74.0	60.0	0.003	65.0	67.1	NS	NS	64.1	68.5	NS
A telephone hotline for supporting	818	90.4	91.5	93.9	88.5	90.4	NS	94.0	86.5	89.8	0.016	90.5	89.5	NS	0.023	89.9	91.7	NS
Reporting through administration and sales systems	758	83.9	75.9	86.4	82.1	84.9	NS	85.7	83.4	83.3	NS	83.8	84.2	NS	0.001	83.8	84.3	NS
Simplifying the reporting process	835	92.5	88.1	95.5	92.9	92.4	NS	91.7	94.5	92.3	NS	92.7	91.4	NS	< 0.001	92.6	92.5	NS
Clear instructions for what to report	840	92.8	93.2	95.5	91.7	92.8	NS	93.0	92.0	93.0	NS	92.9	92.1	NS	0.003	92.4	93.8	NS
Clear instructions for how to report	839	93.2	93.2	92.4	91.7	93.7	NS	94.4	92.9	92.5	NS	93.3	92.6	NS	0.025	93.2	93.4	NS
IT access in the pharmacy	816	90.4	91.5	93.8	87.1	90.8	NS	90.3	93.0	89.3	NS	90.5	89.3	NS	NS	90.5	90.2	NS
Mean ± SD: *n* = *777.5* ± *86.6* *%* = *86.1* ± *9.0*																		
**C. Motivations**																		
Regular reminders about reporting	836	92.4	89.8	90.9	89.1	93.6	NS	90.4	92.5	93.8	NS	92.9	89.5	NS	NS	92.1	93.0	NS
Educating patients to recognize and self-report ADRs	867	95.8	88.1	100	92.9	96.8	0.001	95.0	96.0	96.3	NS	95.6	96.7	NS	0.038	94.7	98.8	0.007
Knowing what happens after reporting a case	841	92.9	89.8	93.9	92.3	93.2	NS	90.0	94.0	94.5	NS	92.5	94.7	NS	NS	92.6	93.8	NS
Legal obligation to report ADRs	739	82.1	83.1	92.3	78.7	81.7	NS	87.7	86.0	76.4	< 0.001	80.5	90.7	0.003	< 0.001	79.0	90.4	< 0.001
Remuneration for reporting	545	60.4	62.7	60.6	62.8	59.7	NS	65.1	67.3	53.1	< 0.001	58.3	70.4	0.006	0.041	59.8	61.8	NS
Non-financial incentives for reporting	755	83.4	79.7	80.3	83.3	84.1	NS	84.1	89.5	79.9	0.010	83.2	84.2	NS	NS	83.7	83.1	NS
Colleagues’ interest in reporting	826	91.9	88.1	90.9	91.0	92.7	NS	92.9	95.5	89.3	0.023	91.6	94.0	NS	0.007	91.5	92.9	NS
Clarification of the importance of the pharmacists’ role to the public via the media	864	95.6	91.5	98.5	94.9	95.8	NS	94.7	98.0	95.0	NS	95.9	94.7	NS	NS	96.4	93.4	NS
Mean ± SD: *n* = *784.1* ± *107.6* *%* = *86.8* ± *11.6*																		

*ADR* adverse drug reaction, *CPD* continuing professional development, *EPS* Egyptian Pharmacists Syndicate, *IT* information technology, *NS* non-significant

^a^ Valid percentage of respondents who answered positively

^b^ Valid percentage of respondents who answered positively within the related group

^c^ Pearson’s chi-square test. Significance at *p* ≤ 0.05

**Table 6:** Differences in adverse drug reaction reporting preferences and training status versus the facilitators among pharmacists (N = 905)

Facilitators	n	(%)^a^	Have reported^b^			Prefer paper reporting (Yellow Card) (%)^b^			Prefer electronic reporting (%)^b^			Received ADR reporting training^b^		
			No	Yes	*P*^*c*^	No	Yes	*P*^*c*^	No	Yes	*P*^*c*^	Male	Female	*P*^*c*^
**A. Educational intervention**														
University teaching about the reporting process	866	95.7	95.6	96.0	NS	95.6	96.0	NS	95.0	97.1	NS	95.2	96.4	NS
University teaching about ADRs	876	97.0	97.0	96.8	NS	97.4	95.4	NS	97.8	95.3	0.047	97.0	96.9	NS
CPD delivered by the EPS	832	91.9	92.4	88.8	NS	93.0	87.3	0.013	92.2	91.3	NS	92.3	91.4	NS
CPD delivered by the health directorate	766	84.7	85.4	80.8	NS	86.3	78.5	0.01	84.5	82.6	NS	84.5	85.0	NS
CPD delivered by a university	752	83.2	82.8	85.6	NS	84.4	78.6	NS	84.2	80.5	NS	85.8	79.4	0.012
CPD delivered by pharmaceutical companies	788	87.1	86.8	89.6	NS	86.6	88.3	NS	84.4	92.9	0.001	85.3	89.7	NS
Peer-reviewed online journal articles	820	90.6	90.6	90.4	NS	90.2	92.5	NS	89.6	92.8	NS	88.6	93.6	0.011
Mean ± SD: *n* = *814.3* ± *47.8* *%* = *90.0* ± *5.3*														
**B. The reporting process**														
A smartphone application for reporting	724	80.0	79.1	85.6	NS	79.9	80.3	NS	79.7	80.9	NS	79.6	80.6	NS
Easily accessible Yellow Cards	590	65.3	64.5	70.7	NS	65.2	65.7	NS	64.9	66.7	NS	65.8	64.7	NS
A telephone hotline for supporting	818	90.4	91.2	85.6	NS	91.1	87.9	NS	91.0	88.8	NS	90.8	89.7	NS
Reporting through administration and sales systems	758	83.9	83.4	87.1	NS	84.1	83.1	NS	82.9	86.2	NS	83.0	85.2	NS
Simplifying the reporting process	835	92.5	92.7	91.2	NS	92.1	94.2	NS	91.7	94.6	NS	91.9	93.3	NS
Clear instructions for what to report	840	92.8	92.9	92.0	NS	93.3	90.8	NS	92.3	93.9	NS	93.6	91.7	NS
Clear instructions for how to report	839	93.2	93.7	90.2	NS	93.8	90.6	NS	93.4	92.7	NS	93.9	92.2	NS
IT access in the pharmacy	816	90.4	90.4	90.3	NS	91.2	86.6	NS	90.4	90.2	NS	90.6	89.9	NS
Mean ± SD: *n* = *777.5* ± *86.6* *%* = *86.1* ± *9.0*														
**C. Motivations**														
Regular reminders about reporting	836	92.4	92.7	90.4	NS	92.3	92.5	NS	92.6	91.7	NS	92.3	92.5	NS
Educating patients to recognize and self-report ADRs	867	95.8	95.6	96.8	NS	96.3	93.6	NS	95.7	96.0	NS	95.0	96.9	NS
Knowing what happens after reporting a case	841	92.9	93.1	92.0	NS	93.6	90.2	NS	93.66	91.3	NS	92.6	93.3	NS
Legal obligation to report ADRs	739	82.1	81.8	84.0	NS	81.7	84.2	NS	81.5	83.6	NS	79.8	85.5	0.028
Remuneration for reporting	545	60.4	59.3	68.0	NS	59.7	63.6	NS	60.6	59.8	NS	60.9	59.6	NS
Non-financial incentives for reporting	755	83.4	83.6	82.4	NS	84.0	80.9	NS	81.9	87.0	NS	83.1	84.2	NS
Colleagues’ interest in reporting	826	91.9	91.6	93.6	NS	91.2	95.4	NS	90.8	94.2	NS	90.1	94.4	0.022
Clarification of the importance of the pharmacists’ role to the public via the media	864	95.6	95.6	95.2	NS	95.3	96.5	NS	95.2	96.4	NS	95.2	96.1	NS
Mean ± SD: *n* = *784.1* ± *107.6* *%* = *86.8* ± *11.6*														

*ADR* adverse drug reaction, *CPD* continuing professional development, *EPS* Egyptian Pharmacists Syndicate, *IT* information technology, *NS* non-significant

^a^ Valid percentage of respondents who answered positively

^b^ Valid percentage of respondents who answered positively within the related group

^c^ Pearson’s chi-square test. Significance at p ≤ 0.05

#### Improvement of the reporting process

Enhancing the reporting process itself was deemed necessary, including clear instructions on what (840, 92.8%) and how (839, 93.2%) to report, and simplifying the reporting process (835, 92.5%) (Tables 5B and 6B). The preferred reporting methods were via a smartphone application (724, 80.0%) and the pharmacy electronic operating system (758, 83.9%). The availability of support through a telephone hotline was also required (818, 90.4%).

#### Factors motivating reporting

The preferred motivations included regular reminders about reporting (836, 92.4%), promoting the pharmacists’ role via the media (864, 95.6%), and educating patients to recognize and self-report ADRs (867, 95.8%) (Tables 5C and 6C). The legal obligation to report was acknowledged (739, 82.1%) with significant differences based on position (*P* < 0.001), university of graduation (*P* = 0.003), graduation year (*P* < 0.001), sex (*P* < 0.001), and training status (*P* = 0.028).

## Discussion

**Fig. 2:**
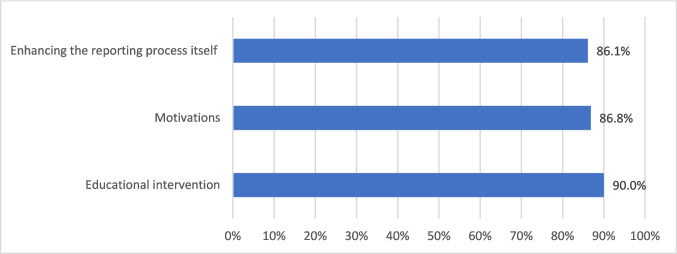
Potential facilitators to adverse drug reaction reporting as suggested by the pharmacists

The participating community pharmacists, registered between 1972 and 2022, ranged in age from 23 to 75 years. Their Lack of vigilance in reporting actual ADRs included underreporting and inadequate characteristics of reported data. This lack could be mitigated by perceived potential facilitators identified across three domains: educational intervention, motivations, and the enhancement of the reporting process itself (Fig. 2).

The following discussion places the findings of the present study in the context of suggesting situation-specific policy amendments regarding necessary potential facilitators required to improve the rate and quality of ADR reporting to healthcare authorities by professional community pharmacists in Egypt.

### Case reporting

#### Underreporting

Similar to their important role in dispensing and counseling within pharmacies, ADR reporting by pharmacists is equally crucial to authorities. Given their proximity to customers and their day-to-day involvement in the provision and appropriate use of medicines, pharmacists are well-positioned to report ADRs. However, our study found that only a few pharmacists had reported an ADR on behalf of a customer, indicating that underreporting leads to missed opportunities to improve drug safety. This finding, while presenting an opportunity for improvement, is unsurprising as underreporting is observed across many countries (Hazell and Shakir 2006; Li et al. 2018; Karuppannan et al. 2022).

#### Inadequate characteristics of reported data

Among the small subset of pharmacists who had reported ADRs, about a quarter neither correctly identified ADR types nor received any training on reporting. This indicates the potential inaccuracy of reported data. Unfortunately, there is no available database in the Egyptian system to enable pharmacists to mitigate this knowledge gap (such as the FEARS database provided by the FDA in the USA). Additionally, pharmacists with more recent graduation years were less likely to report ADRs. Unfortunately, the yellow card (ADR reporting form) in Egypt does not include details about reporters, which could lead to potential biases in reporting practices. Similarly, the yellow card in the UK and the blue card in Australia do not include such details. There is variability in the data fields used across different countries (Bailey et al. 2016). The importance of accurate and unbiased reported data extends to the reliability and validity of national figures and surveillance studies, necessitating evidence-based practices from authorities.

### Potential facilitators

#### Educational intervention

Most pharmacists viewed embedding ADR-related teaching within university curricula as a key enabler. Research shows that undergraduates’ knowledge of pharmacovigilance is higher when related learning outcomes, education, and assessment are embedded in curricula, a finding consistent across both developing and developed countries (Zawahir et al. 2015).

Surveyed pharmacists also reported that access to CPD would enhance pharmacovigilance skills. A study found that 21% of Egyptian neurologists agreed that ADR reporting should be compulsory in-service training, highlighting that needs-based CPD is a facilitator across health professions in Egypt and enables multidisciplinary education opportunities (Kopciuch et al. 2022).

In Egypt, CPD is delivered by several different stakeholders (Mohamed Ibrahim 2012). The present study found that, in order of preference, pharmacists opted for CPD to be delivered by the EPS, followed by pharmaceutical companies, the health directorate, and then universities. This preference differs somewhat from developed countries, where CPD provided by pharmaceutical companies or industry is often associated with concerns of commercial bias (Kearney et al. 2019). Professional societies, as not-for-profit organizations, are often seen as best positioned to deliver CPD to their members. Reading peer-reviewed journal articles was also seen as an enabler, though more published research on reporting in Egypt is needed.

#### Improvement of the reporting process

Ongoing knowledge about the reporting process is foundational, even in countries where reporting processes have long been established. A cross-sectional study identified that 60% of pharmacists in the UK agreed that clearer guidance on which reactions to report as a main facilitator of reporting (Hughes and Weiss 2019). In the present work, pharmacists reported facilitators including clear instructions on what and how to report, followed by the simplification of the reporting process. A systematic review highlighted that tedious, lengthy, and awkwardly constructed reporting forms negatively impact reporting (Al Dweik et al. 2017).

Consistent with the digitalization of the healthcare sector, international literature highlights that pharmacists prefer electronic over paper-based reporting. For example, a Turkish study found that 72.6% of professionals chose an online electronic form as their preferred method of reporting (Güner and Ekmekci 2019). In comparison, our study showed that 59.2% of pharmacists who had submitted reports preferred doing so electronically **(**Table 2**)**.

Most pharmacists across all regions of Egypt identified the availably of a smartphone application as a preferred reporting method **(**Table 5c**)**. Egyptian pharmacists can currently submit reports via mail, online, phone call, email, or fax. Many countries have developed mobile-based reporting applications and explored their impact on pharmacovigilance. While results are mixed, they generally show a trend toward low uptake. For example, in the Netherlands, India, and Croatia, the number of ADR reports completed each month per 1,000 application downloads was 1.1, 7.0 and 4.0, respectively (Pierce et al. 2019). In contrast, the application used in the USA showed a ten-fold increase in reporting. The development and evaluation of a smartphone application in Egypt is needed to determine its impact on reporting rates in future research (Pierce et al. 2019).

#### Factors motivating reporting

Surveyed pharmacists across all positions stated that regular reminders would facilitate ADR reporting, consistent with the “nudge” theory of behavior change (Lamprell et al. 2021). Recent research shows that the nudge paradigm for behavior change is being used to align clinical practice with standards. Pharmacists make decisions in time- and resource-pressured environments; thus, regular reminders may nudge them to prioritize and report.

A key facilitator cited by pharmacists was clarification of the pharmacist’s role in ADR reporting via the media. Media advertising could serve as a nudge for both the public and pharmacists. It may enhance public awareness to recognize and self-report ADRs and motivate pharmacists to submit case reports to authorities. The WHO and many local authorities use the media to reach audiences.

Although legal obligation was acknowledged by most pharmacists, significant differences were noted based on socio-demographic factors. Many Egyptian pharmacists are concerned about the legal ramifications and require legal support and indemnification for their reporting activities. This highlights the legal accountability issues surrounding reporting that require further investigation.

### Policy implications

**Fig. 3:**
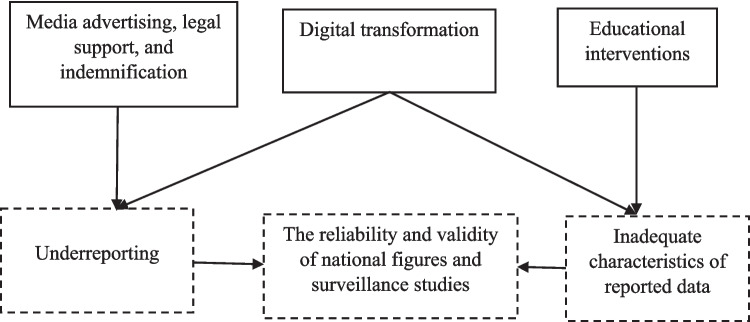
Policy implications

- Educational interventions provided through Universities and the Egyptian Pharmacists Syndicate can improve the accuracy of reported data.
- The media can be used to advertising the importance of ADR reporting, and legal support, and indemnification should be provided to increase reporting rates.
- Clear and continuously updated instructions on the completion of the digital-form, and a simplified reporting process provided using a smartphone application may mitigate both the inadequacy of reported data and the underreporting of ADRs, thus improve national figures (Fig. 3).

### Study limitations and future implications

This study focused solely on Egypt, a low-to-middle-income country. Many findings are country- or socioeconomic status-specific. However, some findings highlight issues common to both developing and developed nations, which could benefit the international research community and policymakers.

Face and content validity of the questionnaire was established by capturing the opinions of twelve highly experienced local pharmacists in addition to conducting a pilot test on a random sample of 36 pharmacies where feedback was requested from the pharmacists regarding the structure and content of the questionnaire. This was deemed sufficient at the time, however those undertaking future studies may want to consider the use of factor analysis.

Future studies investigating the changes in reported metrics following recommended interventions to policymakers (such as educational intervention, new legislation, and reporting tools like a smartphone application) will provide insight into how to enhance reporting practices and consequently improve the reliability and validity of national figures.

## Conclusions

Pharmacists play a crucial role in reporting ADRs to authorities, especially in low-to-middle-income countries, across widely distributed community sites. Pharmacists highlighted that the current reporting practices suffer from both underreporting and inadequate characteristics (inaccuracy and biases) of reported data. These issues can be mitigated by potential facilitators identified in the study. Specifically, educational interventions and improvements in the reporting process itself can simultaneously address both underreporting and data inaccuracies. Media signaling clearly outlining the pharmacist’s role in ADR reporting may also help reduce underreporting.

This study reflects the viewpoints of pharmacy professionals in a real-life context and aims to inform the international research community and policymakers. Future investigations should consider developing targeted education and training materials for reporting, addressing legal accountability issues, and creating a smartphone application for reporting in Egypt. Evaluating the application’s impact on reporting rates will be crucial. Additionally, it is recommended that potential reporters’ details be included in the national ADR reporting form.

## Supplementary Information

Below is the link to the electronic supplementary material.

Supplementary file1 (DOCX 33 KB)

## Data Availability

The dataset presented in this article is available only upon reasonable request since it contains confidential information. Requests to access the dataset should be directed to the corresponding author (ph_hossni@yahoo.com).
