# Supplementary material for: Spontaneous adverse drug reaction reporting: potential facilitators perceived by community pharmacists in Egypt – a cross-sectional study
Source: Saudi Pharm J. 2025 Sep 22;33(5):32. doi: 10.1007/s44446-025-00025-3 (PMC12454801; doi:10.1007/s44446-025-00025-3)
Supplement: Supplementary file 1 — Supplementary file1 (DOCX 33 KB) [file 44446_2025_25_MOESM1_ESM.docx]

**Questions of the survey instrument**

**1. Demographics of community pharmacists and patients’ adverse drug reaction reporting information.**

**A. Community pharmacists**

**1.** What is the region of the community pharmacy? 1.South 2.East 3.Centre 4.North

**2.** What is your position in the pharmacy? 1.Junior 2.Senior 3.Registered manager

**3.** What year did you graduate? ………………

**4.** How many years’ experience do you have? ………………

**5.** What is your gender? 1.Man 2.Woman

**6.** What is your age? ……….. year

**7.** What sort of university did you graduate from? 1.Private 2.Government-funded

**B. Reporting practice**

**1.** Could you correctly identify **all** of the following ADR types? 1.Yes 2.No

(**1.** non-dose-related (Bizarre), **2.** Dose-related (Augmented), **3.** Time-related (Delayed), **4.** Dose-related and time-related (Chronic), **5.** Failure of therapy (Failure), and **6.** Withdrawal (End of use))

**2.** Do you prefer paper reporting (yellow card)? 1.Yes 2.No

**3.** Do you prefer electronic reporting (online)? 1.Yes 2.No

**4.** Did you receive training on ADR reporting? 1.Yes 2.No

**C. Patients of pharmacy complained about ADR**

**1.** What has been the severity of most patients’ symptoms?

1.Mild 2.Moderate 3.Severe 4.Three degrees 5.Mild and moderate 6.Mild and severe 7.Moderate and severe

**2.** What has been the type of the causative drugs? 1.Prescribed 2.Over-the-counter 3.Both

**3.** What has been the source of the causative drugs? 1.Synthesized 2.Natural 3.Both

**4.** What has been the age category of most patients? 1.Adults 2.Children 3.Both

**2. Perceived facilitators for adverse drug reaction reporting.**

**A. Enhancing Pharmacist knowledge and skills**

**1.** Do you need, for enhancing the knowledge and skills, university teaching about reporting process? 1.Yes 2.No

**2.** Do you need, for enhancing the knowledge and skills, university teaching about ADRs as a facilitator? 1.Yes 2.No

**3.** Do you need - as a facilitator- continuous professional education (CPD) delivered by the syndicate as a facilitator? 1.Yes 2.No

**4.** Do you need, for enhancing the knowledge and skills, CPD delivered by the health directorate as a facilitator? 1.Yes 2.No

**5.** Do you need, for enhancing the knowledge and skills, CPD delivered by a university as a facilitator? 1.Yes 2.No

**6.** Do you need, for enhancing the knowledge and skills, CPD delivered by pharmaceutical companies as a facilitator? 1.Yes 2.No

**7.** Do you need, for enhancing the knowledge and skills, peer-reviewed online journal articles as a facilitator? 1.Yes 2.No

**B.** **Pharmacist Motivations** **toward ADR reporting**

**1.** Do you prefer the motivation through regular reminders about reporting as a facilitator? 1.Yes 2.No

**2.** Do you prefer the motivation through educating patients to recognize and self-present with ADRs as a facilitator-? 1.Yes 2.No

**3.** Do you prefer the motivation through knowing what happens after I report a patient as a facilitator? 1.Yes 2.No

**4.** Do you prefer the motivation through legal obligation to report ADRs as a facilitator? 1.Yes 2.No **5.** Do you prefer the motivation through remuneration for reporting as a facilitator? 1.Yes 2.No **6.** Do you prefer the motivation through non-financial incentives for reporting as a facilitator? 1.Yes 2.No **7.** Do you prefer the motivation through see colleagues’ interest in reporting as a facilitator? 1.Yes 2.No **8.** Do you prefer the motivation through promoting of the pharmacists’ role importance to the public via media as a facilitator?

1.Yes 2.No

**C. Improvement of the reporting process itself by local authority**

**1.** Do you identify availably of A smartphone app for reporting as a facilitator? 1.Yes 2.No

**2.** Do you identify Easily accessible yellow cards as a facilitator? 1.Yes 2.No

**3.** Do you identify availably Telephone hotline for supporting as a facilitator? 1.Yes 2.No

**4.** Do you identify availably of reporting through administration and sale system as a facilitator? 1.Yes 2.No

**5.** Do you identify simplifying the reporting process as a facilitator? 1.Yes 2.No

**6.** Do you identify clear instructions for what to report as a facilitator? 1.Yes 2.No

**7.** Do you identify clear instructions for how to report as a facilitator? 1.Yes 2.No

**8.** Do you identify availably of IT access in the pharmacy as a facilitator? 1.Yes 2.No
